# Using interprofessional education to build dynamic teams to help drive collaborative, coordinated and effective newborn care

**DOI:** 10.1186/s12887-023-04373-8

**Published:** 2023-11-15

**Authors:** Josephine Langton, Sara Liaghati-Mobarhan, Edith Gicheha, Jennifer Werdenberg-Hall, June Madete, George Banda, Elizabeth M. Molyneux, the NEST360 Education Team, Ahazi Manjonda, Angela Okolo, Caroline Noxon, Catherine Paul, Charles Osuagwu, Chinyere Ezeaka, Christina Samuel, Danica Kumara, Daphne Flowers, Dolphine Mochache, Ekran Rashid, Emmie Mbale, Esalee Andrade-Guerrero, Evelyn Zimba, George Okello, Georgina Msemo, Grace Irimu, Grace Soko, Harold Chimphepo, Josephat Mutakyamilwa, Karim Manji, Kondwani Kawaza, Maria Oden, Maureen Majamanda, Mustapha Bello, Nahya Salim, Olabisi Odosunmu, Olukemi Tongo, Opeyemi Odedere, Rebecca Richards-Kortum, Robert Tillya, Sara Desai, Steve Adudans, Vincent Ochieng, William Macharia

**Affiliations:** 1 Department of Paediatrics and Child Health, Kamuzu University of Health Sciences, Blantyre, Malawi; 2 Rice360 Institute for Global Health Technologies, Rice University, Houston, TX USA; 3 Texas Children’s Hospital, Houston, TX USA; 4 Kenyatta University, Nairobi, Kenya; 5 NEST360, Kamuzu University of Health Sciences, Blantyre, Malawi

**Keywords:** Interprofessional Learning, Clinical, Biomedical, Sustainable Teaching Materials

## Abstract

**Background:**

As countries strive to achieve sustainable development goal 3.2, high-quality medical education is crucial for high-quality neonatal care. Women are encouraged to deliver in health units attended by a skilled team. Traditionally, the team is doctors and nurses, but they are members of a large group of interdependent experts from other disciplines. Each discipline trains separately, yet the goal of good neonatal care is common to all. The use of interprofessional education breaks down these professional silos improving collaborative practice and promoting excellent clinical care. Introduction of new educational materials and training requires a rigorous approach to ensure sustainability.

**Methods:**

An extensive needs assessment identified gaps in neonatal training. Specifically, there was a lack of inclusion of medical devices used in clinical care. In each country, national key stakeholders came together to develop and revise their own neonatal curricula, trainings or guidelines. A core writing education team were tasked to develop evidence-based materials on pertinent medical devices to include in these national materials. These then underwent internal and external review. A provider course for biomedical engineers and technicians was introduced. Skills labs were established to improve practical skills teaching. To improve the quality of teaching, a NEST360 generic instructors course (GIC) was developed.

**Results:**

Twenty modules, 14 scenarios, 17 job aids and 34 videos have been published to date. Materials have been embedded into neonatal curricula and national trainings. Forty-one skills labs were installed in pre-service learning institutions and, up to June 2022, have been used by 7281 students. Pre- and in-service interprofessional training was implemented at all NEST360 institutions (clinical and biomedical). GIC courses were conducted at least twice a year in all countries. Three hundred seventeen nurses, biomedical and clinical staff have undertaken the GIC in all four countries. GIC participants report that the course has very positively influenced their teaching practice.

**Conclusions:**

Inclusion of key stakeholders throughout has ensured training is embedded within the four countries. Use of interprofessional education and inclusion of biomedical engineers and technicians has been very successful. Introduction of the GIC has developed a pool of high-quality educators for neonatal care. This approach has ensured that high-quality interprofessional neonatal training is included within national agendas for neonatal care and beyond.

**Supplementary Information:**

The online version contains supplementary material available at 10.1186/s12887-023-04373-8.

## Key findings

**Table:** 

**What was known?**
• High-quality education is crucial for delivery of high-quality clinical care • Interprofessional education is important to break down professional silos • A needs assessment is a fundamental part of introducing new educational materials • Medical education is in a perpetual state of unrest • Good healthcare professionals do not necessarily equate to being good teachers
**What was done that is new?**
• Inclusion of all key stakeholders throughout the development process • Expansion of healthcare team to include biomedical engineers and technicians (BME/Ts) within training • Emphasis on interprofessional education • Development of educational materials which were adaptable enough to span both pre- and in-service trainings • Emphasis on ensuring all trainers are appropriately qualified • Robust quality assurance process ensuring high-quality materials produced
**What was found?**
• Inclusion of key stakeholders ensures training is embedded within academic curricula and national training programmes • Breadth of materials produced has increased accessibility and usability of the materials (pre- and in-service) • GIC has positively influenced quality of neonatal training • Broadening the definition of a healthcare team to include biomedical engineers and technicians has significantly increased awareness of and respect for how BME/Ts can contribute to neonatal care with knowledge and maintenance of medical devices in neonatal units • This approach has ensured that high-quality interprofessional neonatal training is included within national agendas for neonatal care
**What next?**
• Long-term evaluations are planned to ascertain whether this approach translates into sustained, improved clinical practice • We plan to publish expanded explanations of experience with: ◦ The Generic Instructors Course (NEST360 GIC) ◦ The role and application of mentorship ◦ Quality improvement initiatives within NEST360 education and training package ◦ Data for action – using data to drive educational activities

## Background

High-quality care is crucial to reduce neonatal mortality and achieve sustainable development goal 3.2 [1]. It requires robust pre- and in-service training, multidisciplinary teamwork, access to appropriate medical devices and professionals able to use and maintain this equipment.

A multidisciplinary team within healthcare is traditionally comprised of different types of clinical staff. However, when medical devices are essential for safe and effective care, such as in a ward for small and sick newborns, it is important to consider expanding the team to include a broader multi-cadre group (e.g., biomedical engineers and biomedical technicians). One of the biggest challenges to broadening multidisciplinary teamwork is that training typically occurs in professional silos [2–6]. This inhibits collaboration, limits understanding of and respect for each other’s roles and drives a hierarchical system within the workplace [2]. As collaborative practice “strengthens health systems and improves health outcomes” [7], it is crucial to break down these divisions. One approach to this is through inter-professional education (IPE) [2].

Undergraduate training (i.e., pre-service) is traditionally uni-professional, and though providing core knowledge, skills and attitudes required to deliver healthcare [8], professional silos are retained. In-service training may be multi-professional, often focusing on a specific topic (e.g., neonatal care), but again maintains the silos. However, in-service training can be inter-professional. The purpose of IPE is “to learn with, from and about each other” [8], thereby breaking down professional divisions. This leads to greater respect for each other and a better understanding of the contribution that each makes to the team, thus improving collaboration [8].

Establishing new educational curricula, training, or material necessitates consultation with and inclusion of all key stakeholders [9]. Performing a needs assessment is crucial to determining educational goals, identifying core content, ascertaining preferred learning formats, and ensuring local relevance. It is also a means of promoting stakeholder buy-in [10]. Learning needs to be contextualised [11], with opportunities for refresher training and ongoing support [12]. As “medical education is in a perpetual state of unrest” [13] it is important to ensure that educational material is adaptable in its format to enable wide integration into national educational programmes. Longitudinal training opportunities are better placed to strengthen communities of practice through use of peer-to-peer learning and mentorship. They improve teamwork and collaboration, with resultant behavioural change and transformation of practice [14]. To cascade learning, many implementers train a large group of participants and select the best to become Trainers of Training (ToT). A ToT course ensures that trainers know the teaching topics; it does not show them how to deliver the training [15].

This paper describes the feasibility and short-term outcomes of the approach used by NEST360 to introduce or revise evidence-based national neonatal training materials, with the inclusion of material on neonatal medical devices. The neonatal device training materials that have been developed are locally owned, adaptable in format, integrated into pre- and in-service training, promote interprofessional learning and have the overall aim of reducing neonatal mortality.

## Methods

### Identifying gaps in country educational materials

In 2019, the NEST360 country education teams (who reviewed all the writing group outputs and led local trainings) undertook a needs assessment in the four partner countries (Malawi, Kenya, Tanzania, and Nigeria) to identify gaps in neonatal training. They evaluated pre- and in-service curricula, training programmes and guidelines for content and scope of neonatal education. The common gap was the lack of inclusion of technologies used to augment clinical care. Nigeria and Tanzania needed to produce comprehensive newborn care guidelines [16, 17] protocols and training programmes. Malawi and Kenya needed to revise existing materials to include current best practices and devices [18, 19]. All four countries had no technical guidelines for the maintenance and troubleshooting of newborn devices.

Key stakeholders for each country (Table 1) were brought together by the local NEST360 education team to develop, update or revise their curricula, training programmes and neonatal guidelines. It was agreed that new materials should harmonise newborn care across each country and would be the basis for developing and accrediting training programmes. All countries agreed on the need to integrate devices into training materials. The NEST360 core education team were tasked to develop educational materials on the pertinent technologies (Table 2), which could then be incorporated into all country training materials.

**Table 1:** Key stakeholders

**Representatives from:**
Academic institutions
Healthcare facilities
Professional societies
Regulatory bodies
Ministry of Health
Ministry of Education
Relevant working groups
Partners working in country
Healthcare staff: clinical, nursing, BME/T

*Abbreviations*: *BME/T* biomedical engineers/technicians

**Table 2:** Devices for which teaching materials were developed

Radiant warmer
Suction machine
Oxygen concentrator
Oxygen flow splitter
Pulse oximeter
Bubble continuous positive airway pressure
LED phototherapy lights (and light-meter)
Glucometer
Bilirubinometer
Syringe pump
Haemoglobinometer
Temperature monitor

*Abbreviations*: *LED* light emitting diode

### Development of NEST360 Education (NESTEd) materials

NESTEd materials were developed by a core interprofessional writing team with long experience in newborn care, education, and communication strategies (Table 3).

**Table 3:** The NEST360 core education writing team

3 paediatricians
3 biomedical engineers
1 neonatal nurse
2 communication experts
1 training materials coordinator

Literature searches were conducted for each device. WHO and United Nations Children’s Fund (UNICEF) recommendations and standards of care, clinical trial findings, systematic reviews, national guidelines, manufacturer user and technical manuals were referenced. Industry experts’ opinions and clinical and biomedical engineering technology (BMET) usability studies were assessed. Through a collaborative writing process, clinical and technical modules for devices were then drafted using a common template (Additional file 1). Colour-coded query and recommendation alert boxes were included to aid decision making. The materials cover devices to enable provision of secondary level care, including provision of continuous positive airway pressure (CPAP) [20]. In accordance with requests from Ministries of Health, these modules were generic to enable use with any available device brand.

**Fig. 1:**
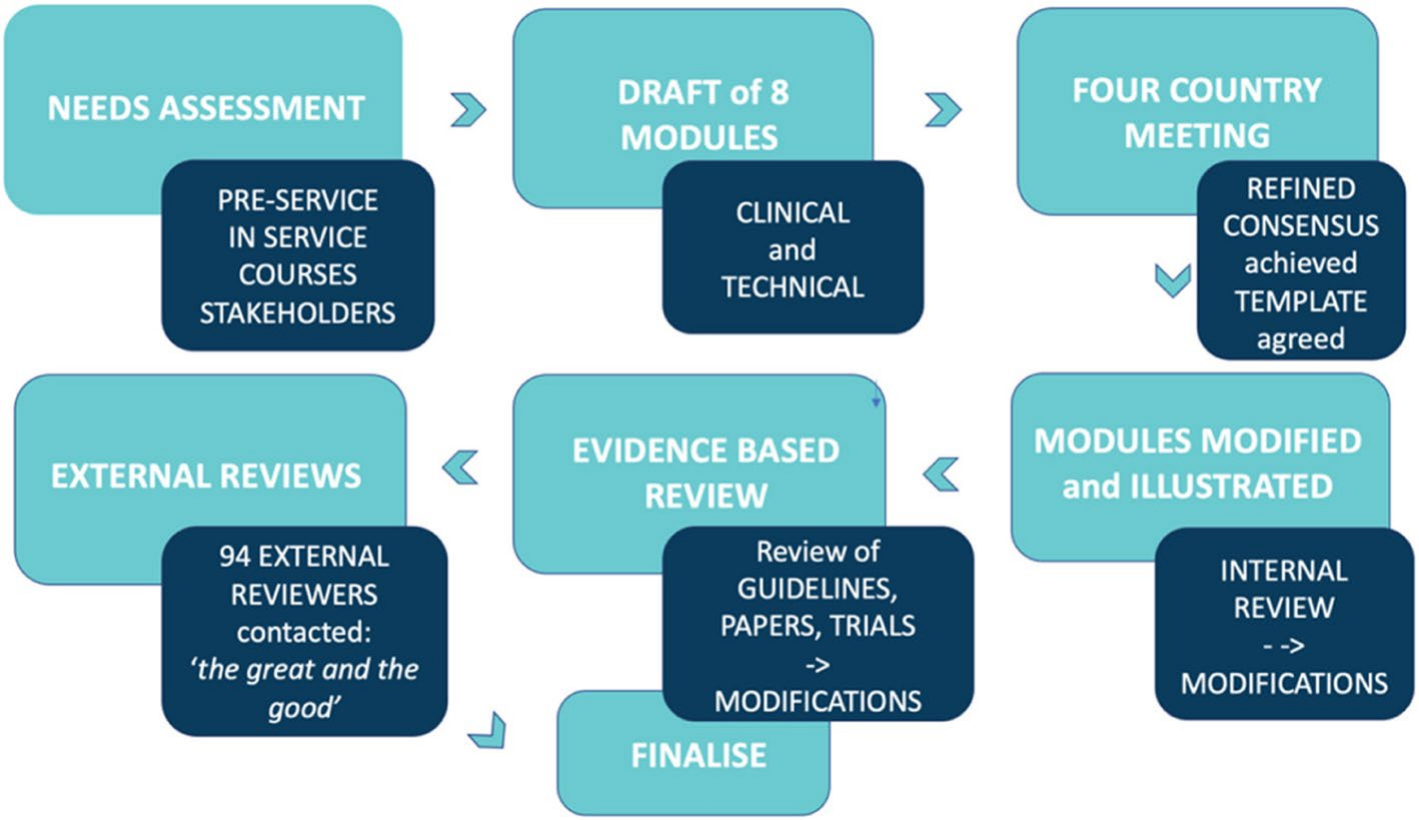
The steps taken in developing NESTEd teaching materials

Module drafts were circulated to the wider country education teams for internal review. Their comments and feedback were documented, moderated, and incorporated as appropriate. Revised drafts were then sent for external expert review. Their feedback was incorporated before publication and circulation (Fig. 1).

Once modules were approved internationally, the core team developed device-specific job aids, videos and presentations. All videos were narrated and demonstrated by staff from countries implementing with NEST360. Clinical and technical scenarios were also written to facilitate training.

### Instructor courses

To ensure the trainers of training could deliver high-quality teaching, a NEST360 generic instructor course (GIC) was developed. GIC materials were adapted from a European model for teaching life support courses [21]. The GIC aimed to enhance skills in lecturing, skills teaching, simulation teaching, facilitating small group discussions and conducting assessments. There were a few plenary lectures, but mostly, the participants were in groups of five or six, in which they each practised various modes of teaching and assessment. Each group had two experienced facilitators. The goal of GIC was to expand the teaching and mentoring skills of clinical and technical professionals, having had the opportunity to ‘try them out’ and gain confidence by practising in small, non-judgmental groups. Examples used for teaching practice in a GIC were based around any common interests of the participants. In our case, the examples used were based on the national neonatal courses. A strength of NEST360 GIC was that biomedical and clinical health workers learn and practice together.

### Skills labs

**Fig. 2:**
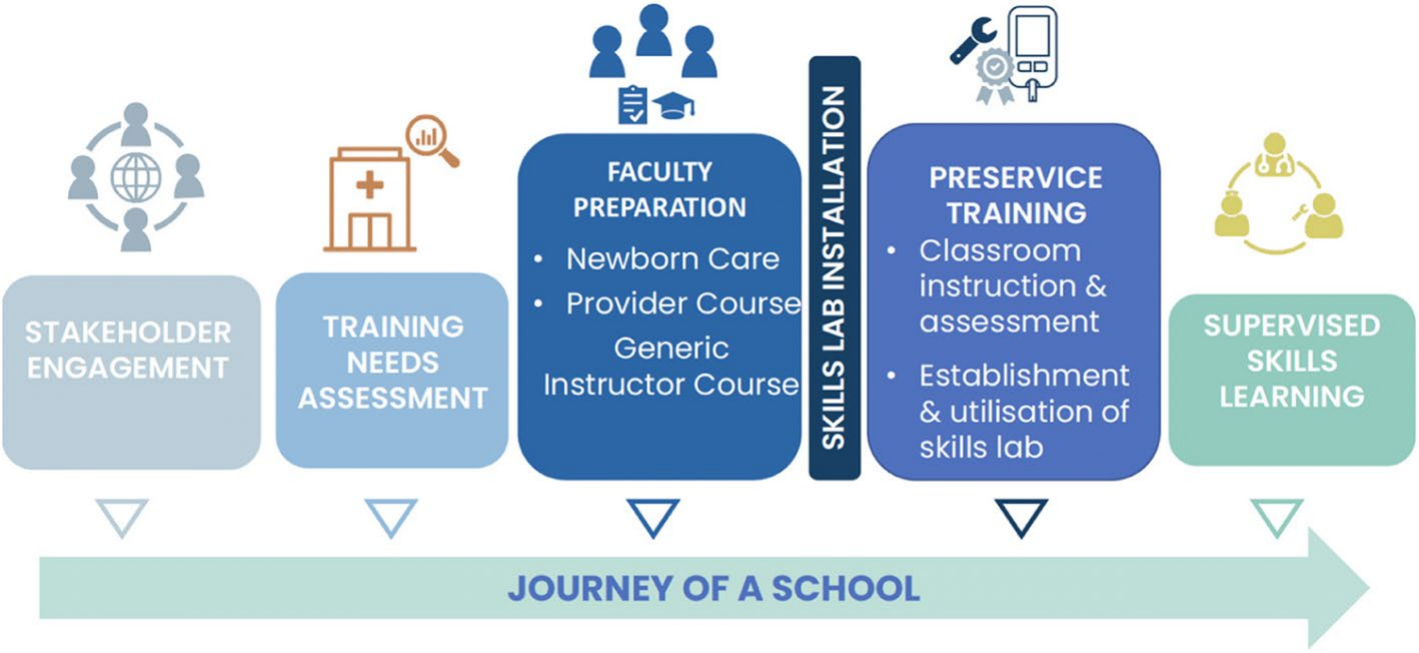
The role and place for a skills lab in a training institution

Recognising the lack of practical skills teaching, NESTEd were requested to establish skills labs with clear guidelines for their use. A process similar to creating the modules was used (Fig. 2). An assessment was made of the number, size and types of practical training facilities available to teaching institutions and hospitals. Equipment in the skills lab and responsible staff for teaching and maintaining the equipment and lab premises in good order were identified. A plan was then made of the number of skills labs needed to be geographically accessible to all students and staff at NEST360-associated institutions. The number and placement of skills labs depended on the number of training centres in the NEST360 supported catchment areas. NEST360 qualified equipment was allocated to each skills lab and installed. During installation, the lab manager and relevant teaching staff were taught how to use and maintain the equipment. Teaching staff were expected to have undertaken a provider course and have attended a GIC course. Teaching materials – modules, job aids, Microsoft PowerPoint presentations and videos – were provided. Lab managers were given a register to record lab usage and to ensure that class teachings did not clash. The skills labs were opened sequentially once basic lab requirements were completed. Post-installation supervision maintained quality and encouraged use.

### NeoTech: a provider course for biomedical engineers and technicians

The Neonatal Technology Management Course (NeoTech), which includes the NEST360 modules, was developed as a five-day provider course to equip biomedical engineers and technicians with knowledge in general use, principles of operation, infection prevention concepts and preventive and corrective maintenance of neonatal care devices. The course involves rigorous practical sessions and participants are evaluated in both a practical and written medium.

## Results

### Use of material

The materials on how to use a device were descriptive and user-friendly, listing specific procedures in a stepwise manner with pictures and drawings where appropriate. This made them easy to follow by all cadres of staff and students. Eleven clinical modules, nine technical modules, six clinical and eight technical scenarios, 17 clinical job aids and 34 short videos have been published to date. They are available in print and electronically under the common creative licence (https://nest360.org/resources/). By June 2022, there had been 2962 downloads overall [22]. Soft copies were hyperlinked to other modules and resources. Countries adapted and integrated these materials into comprehensive newborn care national guidelines that are currently used in national training programmes of all NEST360-associated countries.

### Pre-service training

NESTEd materials and interdisciplinary education were incorporated into pre-service curricula at all clinical, nursing, and biomedical NEST360-affiliated institutions. Teachers from 15 of 29 institutions (11 biomedical engineering technology (BMET) schools and 18 clinical schools) responded to a questionnaire asking how new content had been added to curricula. All clinical schools had added or integrated new materials into their curricula. Of the 11 BMET schools, six added new content, two had integrated the materials into the curricula, and three had taken no action. The most widely used materials were for bubble-CPAP (bCPAP), phototherapy and light-meter.

### Skills lab

Forty-one skills laboratories were installed in pre-service learning institutions to improve and standardise the teaching of practical skills in the care of sick and small newborns (Table 4, Additional file 2).

**Table 4:** Skills labs by country^a^

**Country**	**Number of NESTEd -associated facilities**	**Cadre**	**Number of skills labs**
Kenya	13	Clinical	3
		BME/T	8
Malawi	38	Clinical	19
		BME/T	2
Tanzania	7	Clinical	3
		BME/T	3
Nigeria	11	Clinical	2
		BME/T	1
**Total skills labs installed**			**41**

*Abbreviations*: *BME/T* biomedical engineers/technicians

^a^Data collected in June 2022

**Fig. 3:**
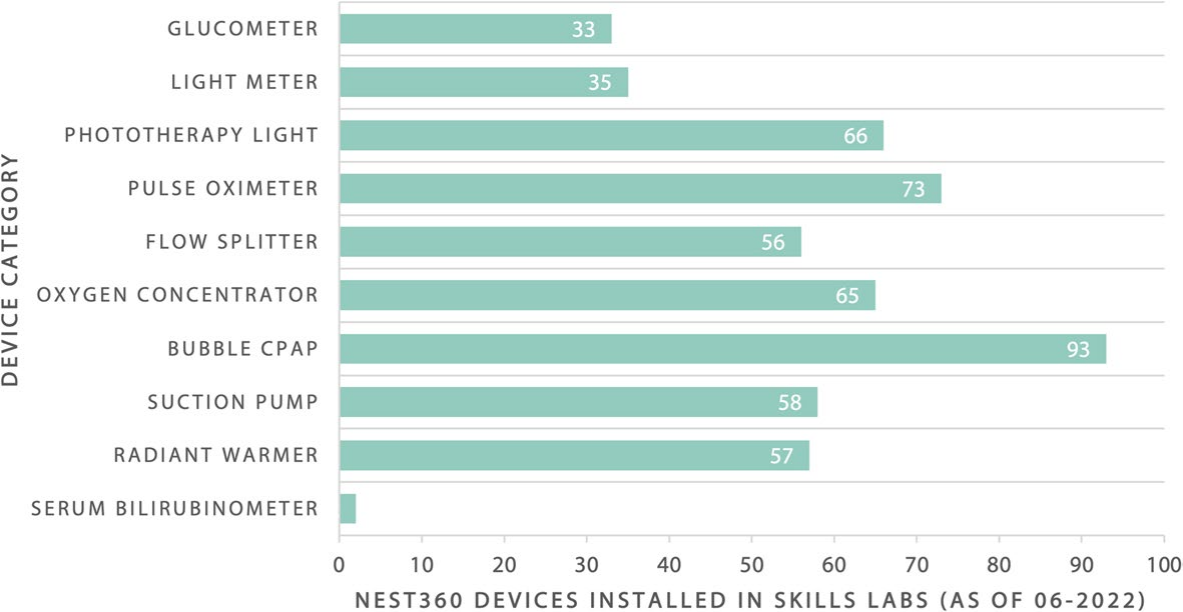
Number and types of devices installed in skills labs

Every NEST360-associated pre-service institution had access to a fully equipped skills lab (Fig. 3) and had at least one trained faculty member to ensure that clinical and biomedical students received both knowledge and necessary practical skills prior to clinical placements in newborn units. Pre-service faculty staff were intentionally chosen to be trained first to enable the dissemination of up-to-date care practices and the integration of updated standards and practical teaching into local curricula.

From June 2020, when training started, until June 2022, the skills labs had been used by 7281 students, mostly second and third-year nursing and biomedical students. Better documentation of lab use is needed as only half (51%) of the schools kept a strict record of who used the skills lab and for what competencies. Over half (*n* = 22/41) of the skills labs were available to hospital staff of newborn units, as most teaching faculty felt that this fostered better partnerships between them and hospital mentors. It also allowed for orientation of new hospital staff.

There were challenges associated with the use of skills labs, including insufficient mannikins; high student to devices ratio during teaching; concerns over device security; lack of trained skills lab managers and inadequate space for large student classes.

### In-service

Interdisciplinary in-service education was implemented at all NEST360-associated newborn units and pre-service clinical and biomedical learning institutions. This involved 38 hospitals in Malawi, 13 in Kenya, 11 in Nigeria and seven in Tanzania. At each site, clinicians and nurses were trained on national newborn care guidelines. Biomedical engineers and technicians (BME/Ts) undertook a biomedical course, and together, they were all taught best practices in the use and maintenance of newborn care devices.

All countries adapted and integrated clinical modules and job aids into their country guidelines. Subsequently, trainings to disseminate the information were organised by and held in each country. Every country trained staff in the facilities where the package of devices was installed. Table 5 records clinical and BMET trainings held and outcomes per country. Pre- and post-training tests showed an increase in knowledge and practical use, ranging from 24 to 41% for both technical and clinical staff.

**Table 5:** Inservice provider course training results^a^

**Country**	**Number of facilities**	**Cadre**	**Total staff trained in facilities**	**Median Pretest Scores (%)**	**Median Post test Scores (%)**	**Improvement (%)**
Kenya	13	Clinical	440	50	83	33
		BME/T	123	61	87	26
Malawi	38	Clinical	270	61	92	31
		BME/T	112	39	80	41
Tanzania	7	Clinical	259	53	82	29
		BME/T	36	32	67	35
Nigeria	11	Clinical	302	48.7	77	28
		BME/T	77	20.9	41.5	20.6

*Abbreviations*: *BME/T* biomedical engineers/technicians

^a^Data collected in June 2022

Most competencies and skills tested showed that immediately following training, all participants had acquired basic knowledge in the use and maintenance of devices. Each country recorded scores of > 80% in the post-test theoretical and practical assessments on clinical and biomedical provider courses. Excellent course trainees were invited to participate in a GIC as potential instructors for future courses and/or as possible mentors.

### Generic instructor course

GIC courses were conducted at least twice a year in all four countries to train local instructors to be able to use different learning techniques that best suit participants and their activities and facilitate long-term behaviour change. It is recognised internationally that GIC facilitator candidates are selected for their knowledge, enthusiasm, and teaching potential. A candidate must successfully teach on two further GIC courses in which a full facilitator mentors and supervises them. A course director is an experienced GIC facilitator who has shadowed a course director before running a course. A total of 317 nurses, biomedical staff, clinical officers, and doctors have taken the GIC course in all four countries. Kenya and Malawi have seven and two fully qualified course directors and 59 and 17 full instructors, respectively. This enables them to run their own GIC courses. As GIC is new to Nigeria and Tanzania, course directors are still in training, but there are four and 19 full instructors, respectively (Table 6).

**Table 6:** Number of participants trained on Generic Instructors Course (GIC)^a^

**Country**	**Total number trained (BME/Ts, nurses, clinical officers & doctors)**	**Course Directors**	**Full GIC Instructors**	**Awaiting GIC2**	**Awaiting GIC1**
Kenya	135	7	39	6	14
Malawi	98	2	8	4	5
Nigeria	53	0	0	4	0
Tanzania	31	0	3	6	10
**TOTAL**	**317**	**8**	**50**	**20**	**29**

*Abbreviations*: *BME/T* biomedical engineers/technicians

^a^Data collected in June 2022

#### Feedback from GIC courses

There is no formal test in GIC, but participants fill out a questionnaire as to what was particularly useful or hard to understand, which parts of the course they enjoyed most and how it would change their practice. All participants enjoyed being with colleagues from different disciplines and learning from each other. The practical sessions were universally popular; how to teach a skill and do assessments were new to many candidates. Only one or two of all the candidates had been previously on any teaching course. Participants reported that the GIC had very positively influenced teaching practice. Some quotes include:

“I learned that I must give of my best. I learnt different teaching modalities to help communicate with non-technical people and especially how to better teach a skill” *Biomedical Engineer, Malawi*

“You made us see teaching in an entirely new way” *Nurse, Tanzania*

“I have been a teacher for many years and now is when am learning how to teach, thank you NEST360 team” *Senior Nurse Tutor, Nigeria*

“I learnt many things including preparing for teaching [setting] that takes away the distractions. I learnt time management. I enjoyed the scenarios, they were fun.” *Clinical Officer, Malawi*

## Discussion

A careful and considered approach was taken throughout the development and introduction of these NESTEd training materials, starting with an extensive local needs assessment, following the six steps suggested by Eliza Medley [23]: 1. What skills do they/we have? 2. Are the skills needed? 3. Has any former training been done? 4. Who needs the training? 5. How accessible is this programme? 6. Is it a success or not? The key gap identified through the needs assessment was lack of educational materials on neonatal devices used to augment clinical care. In addition to identifying educational gaps, this assessment ensured that any training materials developed were culturally suitable and sustainable. Often in resource poor settings, brief educational training courses are created, introduced and delivered by visiting external experts, resulting in a transient ‘parachute effect’ with no long-lasting influence [24, 25]. By undertaking a local needs assessment, we were able to mitigate this risk and ensure contextual and cultural suitability of the training introduced [9, 26]. It also promoted stakeholder buy-in and enabled the core educational team to develop materials that met not only the needs of students and interprofessional healthcare teams but also of healthcare and academic institutions, national policymakers and Ministries of Health and Ministries of Education [10, 27]. The inclusion of all key stakeholders promoted local ownership of the training materials, thereby promoting sustainability [28].

Our materials reflected two key medical educational theories: constructivism and social constructivism. Constructivism builds upon prior clinical knowledge and experience to promote a deeper level of learning and subsequent translation into practice [29]. This was achieved by collaborating with local partners and contextualising materials into the local clinical setting [30]. The overwhelming premise of social constructivism is that learning is reliant upon interaction with others [31]. This is fundamental to medical education as excellent clinical practice is dependent upon learning from one another in a team. Interprofessional learning was, therefore, the basis on which the NESTEd materials were developed. As the gap identified from the needs assessment pertained to medical devices, it was imperative to broaden the team to include biomedical engineers and technicians. The aim is to establish a community of practice where the use of technology is embedded within the clinical setting and learning is shared across disciplines to improve neonatal clinical care [32]. Using educational theory to inform the development process helped ensure the academic quality of the materials [33].

The core writing team comprised a small interprofessional working group with expertise in medical education, neonatal care, and communication strategies. Equally importantly, most of them worked within the clinical and teaching setting where the materials were to be used. This local knowledge ensured the suitability and relevance of the materials produced. Materials were developed through a collaborative writing process with reference to pertinent literature. Production of evidence-based training materials provides quality assurance for both teachers and learners.

Further quality assurance was ensured by having a robust review process. Draft materials were presented to the wider NEST360 education team within all four countries for consensus opinion, again ensuring local relevance. Following the incorporation of their recommendations, the materials were sent for internal review by key stakeholders, including students, educators, and Ministry representatives. The wealth of expertise of local reviewers enriched and improved the materials promoting local ownership and inclusion in local and national trainings and curricula. All these steps should encourage sustained use of the trainings. The final stage of quality assurance was through external expert review. External experts are neutral, bringing international experience and credibility to the process. All these quality assurance measures are important as it is well recognised that high-quality medical education is paramount to successful delivery of high-quality clinical care [34].

The NEST360 education team recognised that the needs for pre- and in-service trainings may be very different. Therefore, a wide variety of materials were developed to ensure educators can access those most suited to their teaching environment. The modules are generic so that the Ministries of Health can adapt them for similar devices. Job aids are device-specific and can be used during mentorship sessions. Simulations are used during national neonatal trainings or within the pre-service setting. Videos can be used for personal revision or incorporated within pre- and in-service trainings. Skills labs enable students to refine their technical use of the equipment [35]. Because the educational materials span the needs of pre- and in-service trainings, they have been accessed, used, incorporated into curricula, and embedded within national training programmes. This adaptability is crucial as the medical field is in a constant state of change as more advances in medical practice evolve [13].

In striving for trainings of excellence, the importance of ensuring all instructors/educators were suitably qualified was recognised. Being an exemplary healthcare provider does not equate to being a good teacher, and it has long been identified that there is a significant gap regarding educational training of health professionals: “Teaching has been the Cinderella of academic medicine, as a stepchild it has garnered little respect in comparison with that accorded its sister tasks of research and patient care” [36]. Teaching is complex and challenging, so it is essential that healthcare providers are equipped with the knowledge and skills to do this effectively [37]. One of the most welcomed and significant educational trainings that NEST360 introduced was the generic instructor course (GIC). Being taught well improves learning, which will, in turn, translate into better clinical practice and improved quality of care.

A further strength of NESTEd materials was the emphasis on interprofessional education. Training in professional silos inhibits the development of interprofessional collaborative practice [38, 39], so to avert the risk of inadequate clinical care, it is paramount that team members understand the expertise, functions, and roles that others bring to the team. The use of IPE has been proven to improve the quality of care, with examples including reduction of infections, reduction of need for supplemental oxygen and reduction in cost of care within neonatal intensive care [40, 41]. Anecdotally the IPE approach adopted by NEST360, where biomedical engineers and technicians are included in the healthcare team, has successfully broken-down professional silos. It has been evident that there is wider appreciation of the expertise and value of skills that all parties bring to the team. There is also a greater understanding of the important role that BME/Ts play in delivering high-quality neonatal care.

Throughout the process, the NEST360 team strived to ensure the sustainability of the trainings. Inclusion of all key stakeholders during material development led to local ownership and inclusion within academic curricula and national neonatal training programmes. Embedding material in this manner and demonstrating the importance of exemplary training has ensured that high-quality interprofessional neonatal training is included within the national agendas for neonatal care of the four countries implementing with NEST360.

A limitation of this paper is that it simply provides a narrative and short-term evaluation of the approach used. A long-term evaluation is planned to ascertain whether our approach translates into long-term behavioural change. Additionally, we plan to publish more papers covering GIC, our mentorship approach, quality improvement initiatives and data for action components of the NEST360 education and training package.

## Conclusion

The approach used by NEST360 in introducing educational training is unique. Including key stakeholders throughout the development process has ensured that the training is fully embedded within the four countries, thereby encouraging sustainability. The breadth of materials produced has increased the accessibility and usability of these materials within both pre- and in-service settings. Inclusion of biomedical engineers and technicians into healthcare teams is novel and has been immensely successful. The IPE approach has increased awareness of what BME/Ts contribute to neonatal care and improved knowledge and maintenance of medical devices in neonatal units.

As countries strive to reduce neonatal mortality and achieve sustainable development goal 3.2, it is important to remember that the foundation of high-quality care is exceptional medical training delivered by skilled medical educators, and this should be reflected within national budgets and in donors’ funding commitments.

## Supplementary Information

**Additional file 1.** Module development generic template.

**Additional file 2.** Skills lab layout.

## Data Availability

Materials are freely available on the NEST360 website under the common creative licence https://nest360.org›resources. Data is available on request to NEST360.org.

## Abbreviations

bCPAP: Bubble Continuous Positive Airway Pressure
BMET: Biomedical engineering technology
BME/T: Biomedical engineers/technicians
GIC: Generic Instructors Course
IPE: Interprofessional education
NeoTech: Neonatal Technical Provider Course
NEST360: Newborn Essential Solutions and Technologies
NESTEd: NEST education
ToT: Training of Trainers
UNICEF: United Nations Children’s Fund
