# Supplementary material for: Using interprofessional education to build dynamic teams to help drive collaborative, coordinated and effective newborn care
Source: BMC Pediatr. 2023 Nov 15;23(Suppl 2):565. doi: 10.1186/s12887-023-04373-8 (PMC10647162; doi:10.1186/s12887-023-04373-8)
Supplement: Supplementary file 1 — Additional file 1. Module development generic template. [file 12887_2023_4373_MOESM1_ESM.docx]

**Additional file 1**

**Module development generic template**

**Module Structure**

NEST-ED Clinical Modules are intended to help healthcare staff and students understand when and how to use devices for newborn care. These modules may be used by teaching institutions to supplement current newborn care curricula or by hospitals, clinical departments, and individuals to update their knowledge and to better facilitate the effective and safe use of newborn care equipment. The structure of each device module is as follows:

CLINICAL PROBLEM

This section describes when a device may be clinically useful. It does not include all the clinical background in making that decision, as this should be covered in country-specific neonatal care protocols & clinical training materials.

ASSESSMENT

This section explains how a device works, and how it may be useful in certain patient care settings.

MANAGEMENT

Step by step preparation for setting up, checking, and using the equipment is described: followed by explanations of how to remove the equipment from a baby when it is no longer needed, how to clean it, and how to store it safely until further need.

INFECTION PREVENTION

In this section infection prevention measures are described for the equipment when in use and instructions on how to disinfect the equipment both during and after use.

COMPLICATIONS

The complications described in this section are those relating to the use of the equipment and do not include all clinical complications that may arise from underlying medical problems. These are beyond the scope of the modules and should be covered in clinical training materials.

CARE & MAINTENANCE

Advice is given on where to place equipment for use, how to safely handle such devices and their consumables, and how to keep them functioning well by using preventive maintenance measures.

TROUBLESHOOTING & REPAIR

This section provides helpful advice on what to check if equipment is malfunctioning on the ward. It is intended to help healthcare staff deal with minor technical difficulties for which there are simple remedies. Detailed machine maintenance is beyond the scope of these modules and is covered in the technical modules that accompany these clinical ones.

ASSESSMENT QUESTIONS

A few questions are attached based on module content.

REFERENCES & ALERTS

References and alert boxes are included within each module to provide clarity on areas where recommendations are governed by published standards, evidence, and/or expert opinion. This is included for the dual purpose of facilitating (1) feedback and continuous improvement of NEST-ED Education Modules and (2) implementer review of content for incorporation in local trainings.

| ** Alert 0.0** Subject |
| --- |
| QUERY ALERT BOXES appear where there may be controversy or disagreement. In these cases, alert boxes provide background to the recommendations that are made in the body of the document. Relevant documents are cited and brief explanation of reasoning for current module content provided. |

| ** Alert 0.0** Subject |
| --- |
| RECOMMENDATION ALERT BOXES appear where there are recommendations based largely on expert opinion or consensus, or to emphasize an important element of care. Relevant documents are cited and brief explanation of reasoning for current module content provided. |
