# Supplementary figures and images for: Using interprofessional education to build dynamic teams to help drive collaborative, coordinated and effective newborn care
Source: BMC Pediatr. 2023 Nov 15;23(Suppl 2):565. doi: 10.1186/s12887-023-04373-8 (PMC10647162; doi:10.1186/s12887-023-04373-8)

**Additional file 2**

**Skills lab layout**


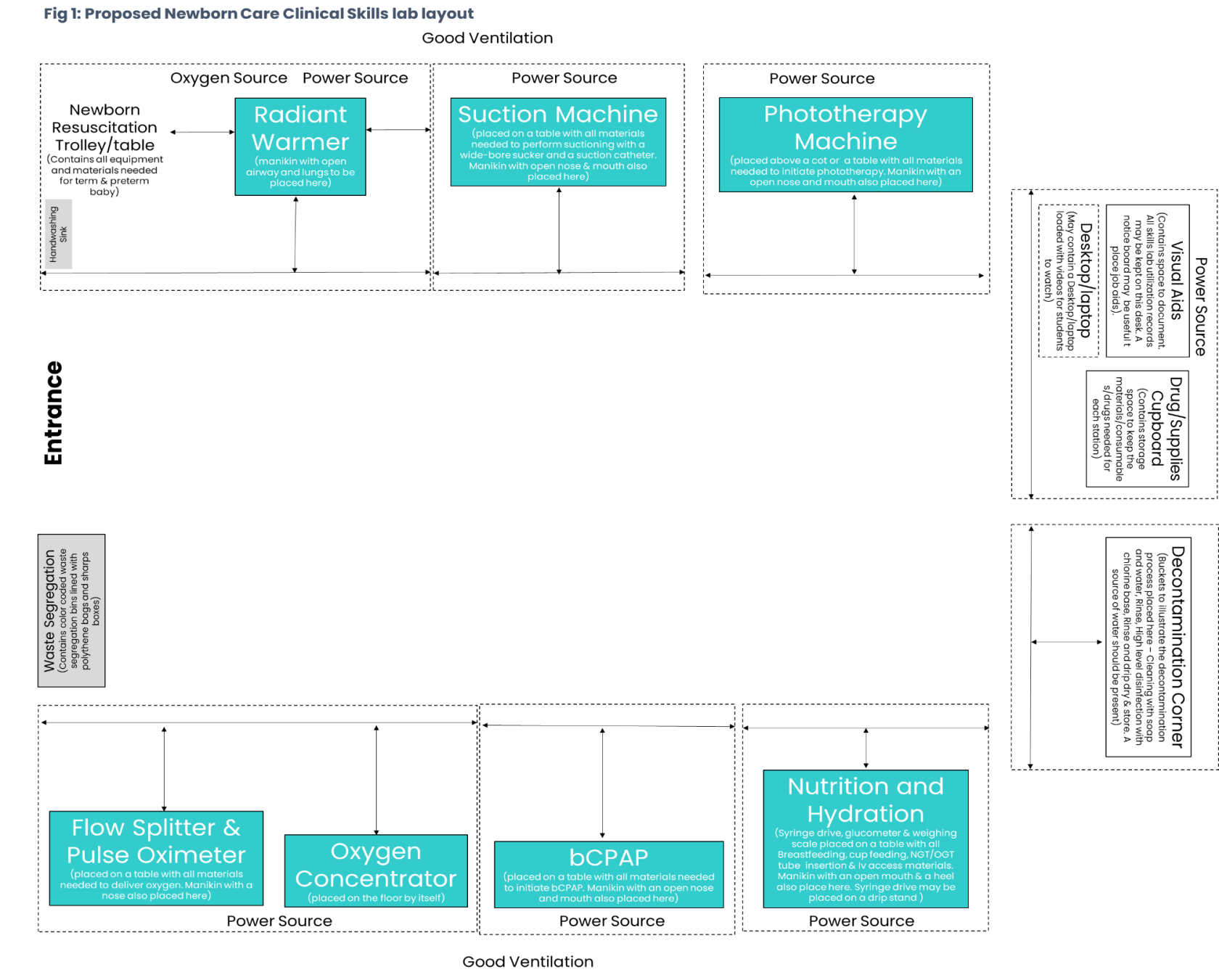

Supplement: Supplementary file 2 — Additional file 2. Skills lab layout. [file 12887_2023_4373_MOESM2_ESM.docx]
